# Identifying Risk Factors for PTSD in Women Seeking Medical Help after Rape

**DOI:** 10.1371/journal.pone.0111136

**Published:** 2014-10-23

**Authors:** Anna Tiihonen Möller, Torbjörn Bäckström, Hans Peter Söndergaard, Lotti Helström

**Affiliations:** 1 Department of Clinical Science and Education, Karolinska Institutet, Stockholm, Sweden; 2 Department of Obstetrics and Gynecology, Stockholm South Hospital, Stockholm, Sweden; 3 Department of Clinical Science, Obstetrics and Gynecology, Umeå University, Umeå, Sweden; Örebro University, Sweden

## Abstract

**Objectives:**

Rape has been found to be the trauma most commonly associated with Posttraumatic Stress Disorder (PTSD) among women. It is therefore important to be able to identify those women at greatest risk of developing PTSD. The aims of the present study were to analyze the PTSD prevalence six months after sexual assaults and identify the major risk factors for developing PTSD.

**Methods:**

Participants were 317 female victims of rape who sought help at the Emergency Clinic for Raped Women at Stockholm South Hospital, Sweden. Baseline assessments of mental health were carried out and followed up after six months.

**Results:**

Thirty-nine percent of the women had developed PTSD at the six month assessment, and 47% suffered from moderate or severe depression. The major risk factors for PTSD were having been sexually assaulted by more than one person, suffering from acute stress disorder (ASD) shortly after the assault, having been exposed to several acts during the assault, having been injured, having co-morbid depression, and having a history of more than two earlier traumas. Further, ASD on its own was found to be a poor predictor of PTSD because of the substantial ceiling effect after sexual assaults.

**Conclusions:**

Development of PTSD is common in the aftermath of sexual assaults. Increased risk of developing PTSD is caused by a combination of victim vulnerability and the extent of the dramatic nature of the current assault. By identifying those women at greatest risk of developing PTSD appropriate therapeutic resources can be directed.

## Introduction

Rape has been found to be the trauma most commonly associated with Posttraumatic Stress Disorder (PTSD) among women [1], and approximately one third of the victims will be diagnosed with PTSD at some time following the assault [2], [3], [4]. PTSD is characterized by persistent re-experiencing of the traumatic event, persistent avoidance of stimuli associated with the event, numbing of general responsiveness, and symptoms of increased arousal [5]. These are all disabling symptoms causing significant impairment in the victim's daily lives, and the burden to the individual as well as to society has been thoroughly reported [6].

Empirical research on risk factors for developing PTSD and the impact of sexual assault has used a broad variety of ways approaching the subject. Many studies tend to divide the numerous potential risk factors into three categories: pre-assault variables, assault variables, and post-assault variables [7]. The pre-assault variables (e.g. demographics, earlier victimization, and psychiatric morbidity) and the assault related variables (e.g. victim-assailant relationship, injuries, perceived life threat) are all fairly easily measurable circumstances that can be used in a clinical setting to identify those victims with highest risk of developing PTSD. The post-assault variables (e.g. social support, coping strategies, and the impact of contact with the legal system) are difficult to assess at an initial acute visit post-rape and therefore are less suitable as predictors in clinical settings. In the current study, performed in a clinical setting, we therefore focus on the first two sets of factors.

Identifying risk factors for the development of PTSD following sexual assault and subsequently permit early interventions with those victims at greatest risk may reduce the incidence [8], [9]. Thus, quite a few studies have been conducted trying to clarify the relationship between various predisposing variables and the occurrence of PTSD after rape, and the literature shows highly discrepant results. Pre-assault victimization has been found in some studies to increase the risk of developing PTSD [10] whereas other studies have not found this association [11]. A majority of the studies have not found any association between different victim-assailant relationships and PTSD [12], [13] however, some have reported that victims of stranger rapes have an increased risk of PTSD compared with victims of other assailants [11], [14], whereas others have found that victims of current partners have the highest risk of developing PTSD [15].

A majority of the conducted studies have a cross-sectional design, often in a large sample of college students, based on the participant’s ability to recall circumstances from past traumatic events. These designs have a risk of causing a non-representative sample with a high proportion of recall bias. Only a few longitudinal studies with acute trauma samples have been conducted and they often suffer from selection bias and problems with too small sample sizes, making generalizations hazardous [2], [4], [10], [16], [17], [18].

The aims of the present study were to: (a) estimate the incidence of PTSD 6 months following a sexual assault using a structured clinical interview, and (b) by using self-report questionnaires at baseline and after 6 months further explore psychiatric morbidity in this population, and (c) prospectively identify factors associated with the development of PTSD at 6 months following a sexual assault. We hypothesized that about one third of the victims would develop PTSD, and that those not diagnosed as having a full PTSD would have major disabling symptoms. We also hypothesized that current PTSD status (i.e. already having PTSD at the time of the index assault) would be higher than in the general population and that victims of sexual assault represent a group with more psychiatric morbidity and history of earlier traumatization. Furthermore, we hypothesized that earlier victimization, psychiatric morbidity, perceived life threat during the assault, and more severe assaults would be predictive of PTSD at 6 months post-rape.

## Method

### Participants

**Figure 1:**
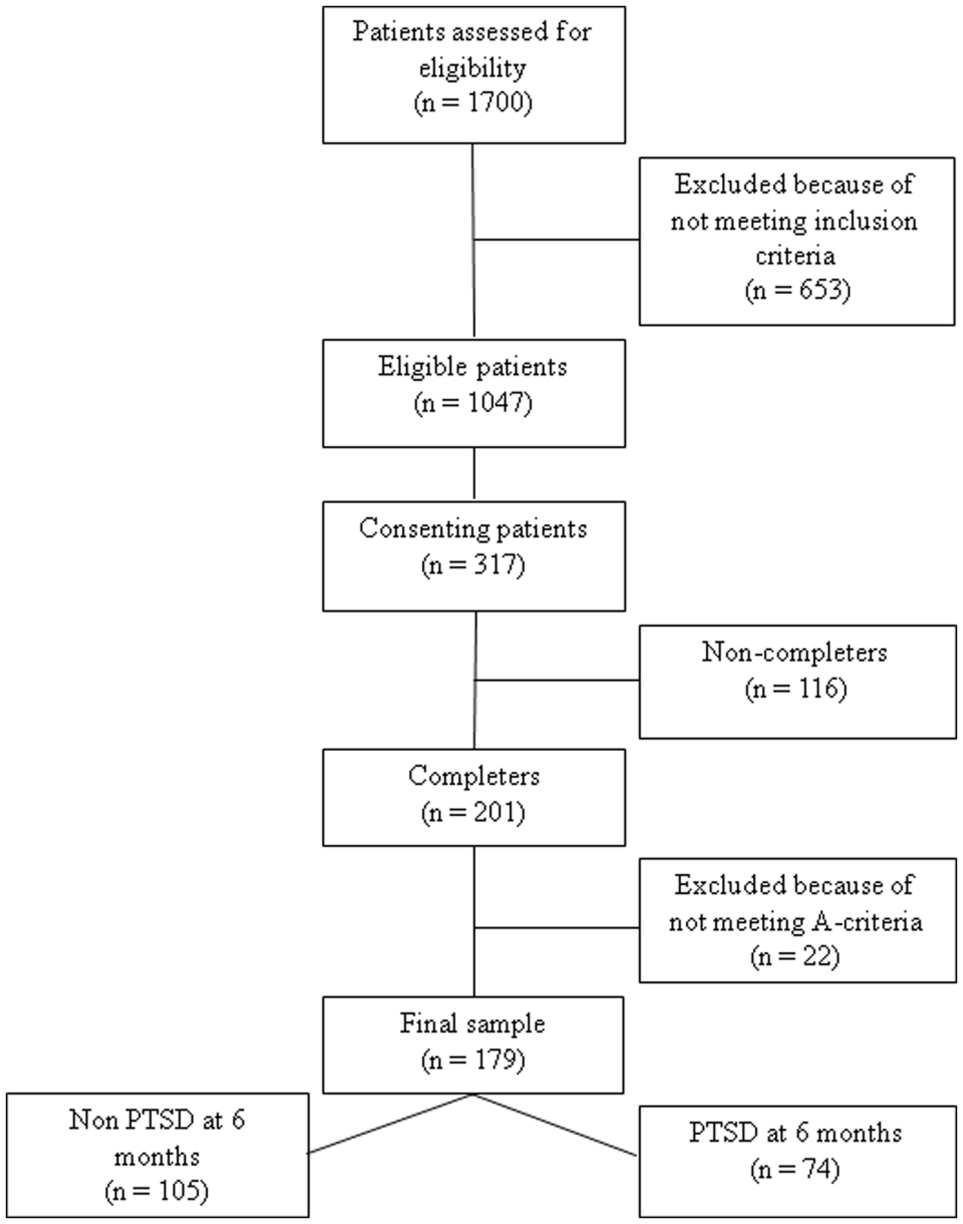
Flow-chart of study population.

Out of 1,047 eligible women, 317 female victims of rape or attempted rape who had been in contact with the Emergency Clinic for Raped Women at Stockholm South Hospital, Sweden, between February 2009 and December 2011 agreed to participate in the study (Figure 1). All women were seen within one month after the index assault. The women were considered eligible if being over the age of 18 years old and literate in Swedish. Further they had to be capable of participating in an interview for diagnosis of PTSD and being able of filling in self rating questionnaires for mental health.

### Measures

The PTSD Module of the Structured Clinical Interview for DSM-IV (SCID I) was used to establish current PTSD 6 months post-rape. The SCID-I is a widely used structured clinical interview [5]. A diagnosis of full PTSD was made using DSM IV-TR (i.e. when clusters A and F were fulfilled).

The level of physical violence used during the assault was defined according to the NorVald Abuse Questionnaire [19] as none, mild (hitting, smacking your face, holding you firmly), moderate (hitting with fist(s) or hard object, kicking, pushing violently) or severe (threat to life, strangulation, showing a weapon or knife).

The Beck Depression Inventory, BDI [20], consists of 21 questions measuring depressive symptoms. Cut-off points used for the sum scores were: 0–9 (no depression), 10–16 (mild depression), 17–29 (moderate depression), and scores ≥30 (severe depression).

The Stanford Acute Stress Reaction Questionnaire, SASRQ [21], was used at baseline and at the 6-month visit post-rape for measuring anxiety and dissociation, and at baseline also for measuring the occurrence of Acute Stress Disorder (ASD). The SASRQ is a 30-item self-report instrument with 3 additional questions relevant to the diagnosis of ASD. The instrument can be used as a Likert-type scale (0–5) or dichotomously (0–2∶0, 3–5∶1) for the presence of a symptom. A diagnosis of ASD according to DSM IV requires at least three out of the five types of dissociative symptoms, one re-experiencing symptom, one avoidance symptom, one marked anxiety/increased arousal symptom, and impairment in at least one important area of functioning. We also used SASRQ total score for measuring PTSD symptom severity.

The Posttraumatic Stress Diagnostic Scale, PDS [22] was used at baseline to assess PTSD symptom score (0–51), pre-existing PTSD diagnosis and lifetime histories of traumatic events. A complete PTSD was diagnosed at baseline when the respondent in PDS part 1 reported having been exposed to or witnessed a traumatic event that according to PDS part 2 involved threat to life or physical integrity and according to PDS part 3 having at least 1 re-experiencing symptom, more than 2 avoidance symptoms, more than 1 arousal symptom, having a duration of mentioned symptoms over 1 month and that the symptoms according to PDS part 4 also caused impairment in the respondents’ daily life in at least one area.

### Procedures

At a medical check-up appointment approximately 10 days after the acute visit, eligible women were asked to participate in the study. Consenting women completed the 3 self-rating questionnaires: BDI, PDS, and SASRQ. Information about history of earlier sexual assaults, sexual assault in childhood, and number of other earlier traumas were taken from the PDS questionnaire. Information on demographics, employment status, psychiatric treatment history, assault characteristics, and possible injuries were gathered from the clinic's structured data files. Six months after the rape, study participants were diagnosed regarding PTSD using the Structured Clinical Interview for DSM-IV (SCID I) and they filled in 2 of the self-rating questionnaires (BDI, SASRQ).

### Statistical analyses

Group comparisons between completers and non completers, PTSD versus non-PTSD participants, were performed using independent t-tests for continuous variables and chi-square tests for categorical variables. The main outcome of the study, the occurrence of PTSD 6 months post-rape, was analyzed using logistic regression. Our model strategy was as follows: First, correlates of PTSD were examined including psychometric variables, victim characteristic variables, and assault characteristic variables in 3 separate regression models. Variables were entered in these models if they were found significant in the crude analyses. Second, a simultaneous multivariate regression model was tested with significant factors from the three domains analyzed to predict the occurrence of PTSD. Variables were considered significant if the Wald test resulted in a p<0.05. The associations are presented as odds ratios (OR) and 95% confidence interval (CI). Hosmer-Lemeshow goodness-of-fit test was used to examine if the model adequately fitted the data (p-value >0.05 suggests good fit) [23]. The ability of ASD to predict PTSD was assessed by calculating the positive predictive value of ASD according to the formula *a/(a+b)*, where *a* stands for the women with ASD that also developed PTSD and *b* stands for the women with ASD that did not develop PTSD [24]. The correlation coefficient between the SCID-I and SASRQ was calculated and is described as the linear correlation (dependence) between two variables, giving a value between +1 and −1 inclusive, where 1 is total positive correlation, 0 is no correlation, and −1 is total negative correlation. All statistical analyses were conducted using the statistical software version SPSS 20.0.

The study was approved by the local medical ethics committee in Stockholm (2008/759-31).

## Results

### Study Sample

The age range was 18 to 59 years (M = 26.36 years, SD = 8.86). The average time between the assault and the initial acute visit was 3.06 days (SD = 5.56 days) and the time from the assault to the initial psychiatric assessment was 19.9 days (SD = 6.7 days). A majority (70.6%) had experienced a completed rape (i.e., including anal or vaginal penetration). Twenty percent had experienced other sexual assaults (including attempts), and 10.1% could not remember what type of assault they had been subjected to **(mainly because of influence of alcohol)**.

### Attrition

Of the 317 women entering the study, 201 women completed the six month assessment. The socio-demographics (age, ethnicity, marital status, and occupation) of non completers were not different from completers (*p*>.05), apart from current alcohol abuse being more common among non-completers (16.4% vs. 7.5%, *p* = .013). Non-completers were more depressed (*M* = 28.63 vs. *M* = 24.75, *p* = .005) and had more avoidance symptoms at baseline (*M* = 22.04 vs. *M* = 20.33, *p* = .018) than completers, but there was no difference in pre-existing PTSD at baseline (19.1% vs. 19.8%, p>.05).

### Psychopathology at 6 months following rape

According to the SCID-I at six months, 36.8% (74/201) of the women met all 6 criteria for PTSD. When women who had not experienced a Criterion A trauma (22/201) were excluded, the PTSD prevalence was 41.3% (74/179). After excluding the 39 women who, according to the PDS questionnaire, had signs of pre-existing PTSD at baseline, 38.6% (54/140) of the women developed PTSD six months post-rape.

Fifty-four percent of the women (97/179) had a high symptom load of B- (re-experience), C- (avoidance), and D- (arousal) symptoms at 6 months and 74.9.% (*n* = 134) met the duration criteria (E). Only 51% (*n* = 101) reported significant impairment in their daily lives (F).

According to Beck Depression Inventory (BDI), 47.5% suffered from moderate or severe depression 6 months after the assault regardless of PTSD status.

### Comparisons between women with and without PTSD 6 months after sexual assault

Women who fulfilled criteria for a full PTSD status (n = 74) using the SCID-I at 6 months (regardless of pre-existing PTSD status at baseline) were compared with those not having PTSD (n = 105) on various psychosocial measures. The women in the PTSD group reported being more depressed both at the baseline assessment and at 6 months post-rape (*p*<.001). They also reported higher PTSD symptom scores from all symptom clusters at baseline according to the SASRQ. The correlation coefficient between SASRQ and the SCID at 6 months was high, (Rho = .721, *p*<.001). A summary of the psychometrics in the two groups is shown in Table 1.

**Table 1:** Psychometrics at baseline of PTSD versus Non-PTSD.

	PTSD		Non-PTSD				
	(n = 74)		(n = 105)				
	M	SD	M	SD	t	OR	95%CI
**BDI**							
Total score	30.1	11.9	23.0	9.9	−4.3	1.06***	[1.03–1.09]
Severe Depression (%)	54.1		26.0			3.36***	[1.78–6.32]
**SASRQ**							
Total score	104.7	22.1	89.6	25.2	−4.1	1.03***	[1.02–1.05]
Dissociation	34.6	8.6	29.5	10.4	−3.5	1.06**	[1.02–1.10]
Re-experience	18.2	7.1	14.2	6.5	−3.8	1.09***	[1.04–1.14]
Avoidance	21.7	5.9	19.9	6.5	−1.9	1.05	[1.00–1.10]
Arousal	22.7	4.6	20.2	6.3	−2.8	1.09**	[1.02–1.15]
ASD Diagnosis (%)	91.8		74.0			3.92**	[1.53–10.06]
**PDS**							
Pre-existing PTSD (%)	27.0		16.5			1.87 *	[0.90–3.89]

*Note.* Independent t-test for continuous variables presented in means (M) and standard deviations (SD). Pearsons chi-test for categorical variables presented in percent. OR = odds ratio. CI = confidence interval. BDI = Beck Depression Inventory. SASRQ = The Stanford Acute Stress Reaction Questionnaire. PDS = The Posttraumatic Stress Diagnostic Scale. *p<.05. **p<.01.***<.001.

Differences between the two groups concerning characteristics of the victim and assault characteristics are shown in Table 2 and Table 3. The only demographic difference between the two groups was that the women in the PTSD group more often reported being unemployed or on sick-leave. More women in the PTSD group reported having a psychiatric treatment history, a history of a lifetime depression, and were more sexually traumatized in childhood than in the non-PTSD group. One out of five of the women in the PTSD group had experienced more than 5 earlier traumas (both sexual assaults and other traumas). Furthermore, the women suffering from PTSD more often reported the current sexual assault as involving more physical violence, full penetration, multiple sexual acts, perceived life threat and more often having been victims of group assault. A smaller proportion of women in the PTSD group reported amnesia in conjunction with the assault. Notable was also that there was no difference in mean number of visits to a therapist at the clinic between the women who developed PTSD and the ones who did not (*M* = 7.6 vs. *M* = 6.6, *p* = .175).

**Table 2:** Victim characteristics of PTSD versus Non-PTSD.

	PTSD	Non-PTSD		
	*(n = 74)*	*(n = 105)*		
			OR	95% CI
**Mean age (SD)**	27.5 (9.7)	25.6 (7.7)	1.03	[0.99–1.06]
**Ethnicity (%)**				
Swedish	78.4	85.7	0.60	[0.28–1.32]
European	9.5	4.8	2.09	[0.64–6.86]
Non European	12.2	9.5	1.32	[0.51–3.42]
**Marital status (%)**				
Unmarried/No partner	68.9	61.0	1.42	[0.76–2.67]
Married/Co-habitor	16.2	21.9	0.69	[0.32–1.49]
**Occupation (%)**				
Working	47.3	54.3	0.76	[0.42–1.37]
Studying	23.0	27.6	0.78	[0.39–1.56]
Sick leave/Un-employed >3 months	28.4	16.2	2.05 *	[1.10–4.23]
**Current alcohol abuse (%)**	6.8	8.6	0.77	[0.25–2.41]
**Psychiatric treatment history (%)**	58.1	36.2	2.45**	[1.33–4.50]
**Life time depression (%)**	36.5	19.0	2.44**	[1.24–4.82]
**History of sexual assault**				
>18 years old (%)	60.8	49.0	1.61	[0.88–2.95]
<18 years old (%)	45.8	30.8	1.90*	[1.02–3.55]
**Number of earlier traumatic events (%)**				
0	20.5	32.7	0.53*	[0.23–0.99]
1	16.4	27.9	0.94	[0.38–2.32]
2	26.0	20.2	2.05*	[0.86–4.89]
≥3	17.8	11.5	2.46*	[1.13–6.60]
>5	19.2	7.7	3.97**	[1.57–11.45]
**History of ≥2traumatic events**	63.0	39.5	2.62**	[1.41–4.85]

*Note.* Independent t-test for continuous variables presented in means and standard deviations (SD). Pearsons chi-test for categorical variables presented in percent. OR = odds ratio. CI = confidence interval. *p<.05. **p<.01. ***<.001.

**Table 3:** Assault characteristics of PTSD versus Non-PTSD group.

	PTSD	Non-PTSD		
	*(n = 74)*	*(n = 105)*		
			OR	95%CI
**Relationship to assailant (%)**				
Partner	13.7	10.5	1.36	[0.54–3.38]
Acquaintance	50.7	49.5	1.05	[0.58–1.90]
Stranger	15.1	25.7	0.51	[0.24–1.11]
Group	17.8	6.7	3.10**	[1.29–8.03]
Amnesia	2.7	7.6	0.34*	[0.07–1.00]
**Victim under influence of alcohol (%)**	68.1	78.1	0.60*	[0.30–0.90]
**Moderate or severe violence during assault (%)**	32.9	18.1	2.32**	[1.15–4.45]
**Perceived life threat (%)**	48.6	29.1	2.30**	[1.23–4.32]
**Penetrating assault (%)**	79.5	67.6	1.85*	[1.20–4.02]
**Multiple acts (%)**	54.8	34.3	2.32**	[1.26–4.29]
**Physical injury (%)**	87.7	69.4	3.11**	[1.43–7.24]

*Note.* Pearsons chi-test for categorical variables presented in percent. OR = odds ratio. CI = confidence interval. *p<.05. **p<.01.***<.001.

### Predictors of PTSD status

Three regression analyses were conducted separately that analyzed psychometric variables, victim characteristics, and assault characteristics. Variables in these regressions were all found significant in crude analysis (see Table 1, 2, and 3). Of the psychometric variables entered (depression, dissociation, re-experience, avoidance, arousal, and ASD), severe depression (*AOR* = 2.75, 95% CI [1.55, 4.52], *p* = .002) and acute stress disorder (ASD) (*AOR* = 2.61, 95% CI [1.14, 6.00], *p* = .031) at baseline were associated with the development of PTSD.

Of the victim characteristics (life-time depression, psychiatric treatment history, history of sexual assault in childhood, history of sexual assault in adulthood, history of ≥2 traumatic events, and employment status), a history of ≥2 traumatic events (*AOR* = 2.02, 95% CI [1.10, 4.15], *p* = .040) and psychiatric treatment history (*AOR* = 2.01, 95% CI [1.05, 3.83], *p* = .034) were associated with the occurrence of PTSD.

Of the assault variables (physical injury, victim-offender relationship, perceived life threat, type of sexual assault, whether the victim had been under the influence of alcohol, and severity of physical violence during the assault), perceived life threat (*AOR* = 2.15, 95% CI [1.01, 3.76], *p* = .044), having been sexually assaulted by a group (*AOR* = 3.84, 95% CI [1.16, 10.69], *p* = .027), having been subjected to several sexual acts (*AOR* = 2.71, 95% CI [1.39, 4.29], *p* = .004), and having been injured (*AOR* = 2.07, 95% CI [1.00, 4.54], *p* = .050) were found to be significant risk factors. Suffering from amnesia was found to be a protective factor for PTSD (*OR* = 0.31, 95% CI [0.07, 0.95], *p* = .038) in the crude analysis; however, it was not so in the adjusted analysis.

The significant predictors from these three initial regressions were then entered simultaneously into a final model predicting PTSD (Table 4).

**Table 4:** Factors associated with PTSD six months after sexual assault (final model).

	B	S.E	Wald	AOR	95% CI
**Sexually assaulted by group**	1.39	0.67	4.33	4.01*	[1.18, 152]
**ASD at baseline**	1.61	0.68	5.56	4.96*	[1.30, 18.6]
**Multiple acts during assault**	1.06	0.38	7.65	2.93**	[1.49, 6.14]
**Physical injury**	0.91	0.45	4.23	2.48*	[1.22, 6.50]
**Severe depression at baseline**	0.82	0.41	3.92	2.33*	[1.03, 5.10]
**History of ≥2 traumatic events**	0.64	0.34	3.61	1.91*	[1.14, 3.72]
**Psychiatric treatment history**	0.66	0.34	3.75	1.93	[1.00, 3.81]
**Perceived life threat**	0.27	0.40	0.44	1.34	[0.60, 3.89]
**Pre-existing PTSD**	0.15	0.53	0.08	0.89	[0.42, 3.33]

Hosmer and Lemeshow Test: 0.86. AOR = adjusted odds ratio. CI = confidence interval.

*p<.05. **p<.01. ***p<.001.

### ASD as a predictor of PTSD

Suffering from ASD shortly after the sexual assault was as mentioned associated with the development of PTSD, both in crude measures (*OR* = 3.9, 95%CI [1.5–10.1], *p* = .001) and in the final model. However, even though a vast majority of the women developed ASD, only 43% of these women continued to develop PTSD. Unlike what has been found after other kind of traumas [25], ASD as a predictor of PTSD after rape has a high sensitivity (92%) however a low specificity (30%) and low positive predictive value (43%).

## Discussion

The major findings of the present study were as follows: (a) with considerations taken to pre-existing PTSD status at baseline 39% of the women developed PTSD 6 months following rape. Furthermore (b) we found that 19% of the study population already suffered from PTSD at the time of the current sexual assault due to earlier victimization. The major risk factors for developing PTSD found (c) were having been sexually assaulted by a group, suffering from ASD shortly after the assault, having been subjected to several acts during the assault, having been injured, and having a history of ≥2 traumatic events.

To our knowledge the present study is one of the largest longitudinal studies performed in a clinical setting. The use of parallel psychometrics (i.e. both self rating questionnaires and clinical interview) to assess PTSD symptoms give strength to the study. Unlike many other studies we used six months as the time for diagnosis of PTSD. This was based on evidence from earlier studies that the number of people with PTSD decreases substantially within the first three months and that symptoms persisting beyond three to six months have a high probability of becoming chronic [1], [4]. Because of its representative sample of sexually assaulted women from a big catchment area our study substantially adds to the literature concerning mental health in the aftermaths of sexual assaults.

Our finding of a PTSD prevalence of 39% is in line with two earlier longitudinal studies from clinical settings [2], [4]. Our results are also consistent with the PTSD prevalence found in two larger cross sectional studies of representative population based samples [3], [26], which further supports the accuracy of our finding. The high proportion of PTSD symptoms found, (e.g. 85% having re-experiencing symptoms), after 6 months even give further proof of the dramatic nature of a sexual assault. As a majority of the victims present with disabling symptoms after 6 months, but only 51% consider themselves as having a significant impairment in their daily lives, the PTSD diagnosis can be regarded as a blunt instrument. The high co-morbidity between PTSD and depression has been explored in numerous earlier studies [27], and is supported also in the current study where patients in the PTSD group were significantly more depressed and had a history of lifetime depression.

The second finding in our study that one out of five women had a pre-existing PTSD at the time of the current sexual assault is interesting, yet not surprising. We know that earlier trauma substantially increase the risk of re-victimization [10], [28]. Half of the women in the current study population had a history of at least one earlier sexual assault and as much as one third had experienced a sexual assault before the age of 18. These findings support our second hypothesis that victims of sexual assaults differ from the general population with regard to earlier victimization and psychopathology. Having a pre-existing PTSD at the entrance of the study was not strongly associated with having PTSD at the 6 months assessment. Almost half of the women with PTSD at baseline had recovered at 6 months, suggesting an ongoing recovery process. This finding is also in line with the finding from an earlier study [29] that PTSD in response to prior trauma was not predictive of PTSD following subsequent trauma.

The main focus of the present study was to identify the factors associated with the development of PTSD at 6 months following a sexual assault. There was an alarmingly high occurrence of psychopathology found shortly after the sexual assault with almost 80% of the women suffering from ASD at the 2 weeks assessment. The use of ASD to predict development of PTSD after various traumatic events have been supported in earlier studies [30], however the use of ASD as a predictor of PTSD after sexual assaults has been questioned [2]. We found the presence of ASD being highly associated with development of PTSD even in the final regression model. Even though majority of the victims develop ASD, far from all will continue developing PTSD, suggesting a substantial ceiling effect. Thus, ASD is not an optimal predictor of PTSD in samples of rape victims.

In the present study dissociation was found a significant risk factor for PTSD in univariate analysis, however, the association did not remain significant in the multivariable model. This finding was consistent with another study [17] who stated that dissociation was a poor predictor of PTSD in rape victims however a useful predictor after other criminal assaults.

Out of the victim characteristic variables entered in the regression model earlier victimization remained significant. In this study we saw an almost linear effect between the number of earlier traumas and the risk of developing PTSD. As two recent studies have suggested [10], [31], the link between childhood sexual abuse and PTSD seems to be mediated through the increased risk of adult sexual abuse. This further suggests a cumulative effect of traumas. Contrary to some studies [28], [32] current alcohol abuse was not found related to PTSD in the present study. This could be explained by the high number of women with current alcohol abuse among the non-completers.

When exploring the assault variables, the only factors that remained associated with PTSD in adjusted analyses were having been subjected to several acts, having been sexually assaulted by a group and having been injured during the assault. The association between having been subjected to several acts is concordant with another study [33] that found that victims of several rape types were more prone to develop PTSD. In the same line sexual assault by a group probably can be looked upon as a more severe interpersonal violence. In the current study women sexually assaulted by a group had the highest risk of developing PTSD out of the different perpetrator categories. Surprisingly, sexual assaults by single strangers did not increase the risk. Those studies reporting victims of stranger assaults being more prone to develop PTSD [11], [14] however have not discriminated between stranger assaults by a single or multiple assailants, having done so the results might not have been so distinct. In the present study women sexually assaulted by a partner developed PTSD more often than women objected to acquaintance and single stranger assaults. This supports our findings from an earlier study [34] that sexual assault by a partner is even more violent than assaults by strangers.

There are some clinical implications of our findings. By focusing on potential risk factors for PTSD, easily assessed in a clinical setting (e.g. factors already known at the time of the first acute visit) they can be used even in small clinics where psychological treatment is not a matter of course for everyone. With increased knowledge about the largest risk factors for PTSD these clinics more easily can identify those women at greatest risk and resources could be directed to these women. “High risk” women could be referred to specialized crisis centers more quickly and maybe some cases of development of PTSD can be avoided.

The present study has some limitations that need to be mentioned. The fact that almost 37% of the consenting patients were lost to follow-up is of course a potential bias. However, the proportion of women by victim-assailant relationship were the same for the completers in this study as the consecutive patients that sought help during the 13 months evaluated in another study from the same clinic [34], suggesting that our sample was representative for the Stockholm area. In our attrition analysis completers did not differ from non-completers regarding victim- and assault characteristics apart from non-completers more often had a current alcohol-abuse. If this is caused by simple forgetfulness, shame or other factors is unknown. Apart from non-completers being more depressed at baseline, they did not tend to score higher on the psychometrics at baseline compared to the completers. The only PTSD symptom cluster found significantly increased among the non-completers was the avoidance symptoms which could explain the attrition. However, our results might still be biased by not having any information on women who did not seek any help. One could speculate that the rape victims that do not seek care are in some aspects similar to the ones that did not complete the 6 months assessment.

## Conclusion

The overall finding confirms that sexual assault represents one of the most traumatic experiences a person can be exposed to regarding the high incidence of PTSD in its aftermath compared to other traumas [1]. Further, the mean age among the women in this study suggests that sexual assault is something that strikes women in the middle of their development into adulthood. To identify those women at greatest risk of developing PTSD we suggest health workers to identify those women with a potential weakness in form of earlier victimization and pre-existing mental health conditions. Because of the ceiling effect, ASD alone is not enough since far from all would develop PTSD. Therefore a combination of ASD and other risk factors should be looked for. Although certain specific variables may vary between studies they all point towards the same direction; increased risk of developing PTSD is caused by a combination of victim vulnerability and the extent of the dramatic nature of the current assault. Future studies of secondary preventions focusing on these women would be of interest.
